# The Enthalpies of Mixing of Liquid Ni-Sn-Zn Alloys

**DOI:** 10.1007/s11669-014-0288-8

**Published:** 2014-02-22

**Authors:** Yu. Plevachuk, A. Yakymovych, S. Fürtauer, H. Ipser, H. Flandorfer

**Affiliations:** 1 Department of Metal Physics, Ivan Franko National University, Kyrylo and Mephodiy Str. 8, Lviv, 79005 Ukraine; 2 Department of Inorganic Chemistry (Materials Chemistry), University of Vienna, Währinger Str. 42, 1090 Wien, Austria

**Keywords:** calorimetry, enthalpy of mixing, metallic alloys, ternary

## Abstract

The partial and integral enthalpies of mixing of liquid ternary Ni-Sn-Zn alloys were determined. The system was investigated along two sections *x*_Ni_/*x*_Sn_ ≈ 1:9, *x*_Ni_/*x*_Sn_ ≈ 1:6 at 1073 K and along two sections *x*_Sn_/*x*_Zn_ ≈ 9:1, *x*_Sn_/*x*_Zn_ ≈ 4:1 at 873 K. The integral enthalpy of mixing at each temperature is described using the Redlich-Kister-Muggianu model for substitutional ternary solutions. In addition, the experimental results were compared with data calculated according to the Toop extrapolation model. The minimum integral enthalpy of approx. −20000 J mol^-1^ corresponds to the minimum in the constituent binary Ni-Sn system, the maximum of approx. 3000 J mol^-1^ is equal to the maximum in the binary Sn-Zn system.

## Introduction

Low temperature soldering is one of the key technologies for the production of electronics devices. Recently, several types of new lead-free Sn-based solders have attracted the attention of the electronics industry.[1-4] Eutectic or near eutectic Sn-Zn alloys, which have been recognized as possible solder candidates due to their low melting temperatures and low costs, are among them.

The melting point of the eutectic Sn_85.1_Zn_14.9_ solder is 472 K, which is close to that of the conventional Sn-Pb eutectic alloy (456 K) but lower than those of other Sn-based eutectic alloys that are already used in soldering, i.e. Sn-Cu (500 K), Sn-Ag (494 K) or Sn-Ag-Cu (490 K). While the Sn-Zn eutectic alloy has excellent properties as a low temperature solder, it has also some drawbacks. Damage by heat exposure and corrosion in humidity, inferior wettability, easy oxidation and micro-void formation have been encountered to limit the practical use of this solder.[5]

It is known that the poor oxidation resistance of the Sn-Zn eutectic alloy is due to zinc oxidation which occurs both in the primary crystallization of (Zn) and the eutectic phase. If the amount of the (Zn) phase in the Sn-Zn eutectic alloy can be reduced or fixed by formation of intermetallic compounds, it is expected that the oxidation resistance can be improved.[6] Therefore, much research was focused on the addition of alloying elements, such as Cu, Ni, Ag, Sb, or Bi,. Among them, Ni has been considered as a suitable alloying element in lead-free solders due to the formation of stable Ni-Zn binary phases as well as by improving the wettability.[7] Furthermore, the addition of Ni effectively enhances the formation of additional ternary intermetallic compounds which can improve the mechanical properties.[8]

The development of lead-free solders requires a clear and thorough understanding of their structural and thermodynamic properties. The increasing influence of computational modelling in all technological processes generates an increased demand for accurate thermodynamic information for the materials systems involved, which are used as fundamental inputs for any model. The solidification process of a liquid alloy has a profound impact on the structure and properties of the solid material. Therefore, knowledge of the basic properties of the molten alloys prior to solidification becomes very important for the development of materials with predetermined characteristics.

In this work the enthalpy of mixing of Sn-based liquid Ni-Sn-Zn alloys was investigated at 873 and 1073 K. The data obtained are useful for modelling of interatomic interactions of the components as well as for a thermodynamic assessment of the Ni-Sn-Zn system. The experimental data were fitted on the basis of an extended Redlich-Kister-Muggianu model[9] and compared to data calculated according to the Toop extrapolation model.[10]

## Literature Survey

### The Sn-Zn Binary System

Thermodynamic properties of liquid Sn-Zn alloys have been investigated repeatedly.[11-20] The authors used different methods to determine the enthalpy of mixing: calorimetric investigations were carried out in Ref 11-15, the authors of Ref 16-19 used the emf method, and quantitative thermal analysis was used in Ref 20. An endothermic behavior of Δ_Mix_ *H* has been revealed over the whole concentration region with a maximum point at about 65 at.% Zn; only Kleppa reported a temperature dependence of the integral enthalpy of mixing.[11] A critical review of the experimental enthalpy of mixing data was published by Lee.[21]

### The Ni-Sn Binary System

The enthalpy of mixing of liquid Ni-Sn alloys was investigated experimentally in Ref 22-24. According to Haddad et al.,[22] the enthalpy of mixing does not depend on temperature between 867 and 1579 K while such a dependence was reported in the temperature range 1660-1775 K by Lück et al.[23]

A strong temperature dependence of the limiting partial enthalpy of mixing for Ni in Sn was observed by Flandorfer et al.[24] The authors concluded also a certain temperature dependence of the integral enthalpy of mixing in the liquid state near the liquidus curve. Several thermodynamic assessments including phase diagram calculations, based on experimental data, were carried out in the past.[25-27]

### The Ni-Zn Binary System

Experimental data on the thermodynamic properties of liquid Ni-Zn alloys are scarce in the literature[28,29]; all investigations point to an exothermic mixing behavior. The minimum point, shifted to the Zn-reach side from the equiatomic concentration, can be explained by the existence of short-range order in the liquid corresponding to the rather stable γ-phase in the solid state. All thermodynamic optimizations were based on the same experimental data.[30-33]

### The Ni-Sn-Zn Ternary System

No calorimetric data for liquid Ni-Sn-Zn alloys have been reported up to now. However, Gandova et al.[36] attempted an extrapolation from binary thermodynamic data to obtain Gibbs energy values for ternary liquid alloys, using different geometrical models as well as the CALculation of PHAse Diagrams (CALPHAD) method. Various groups of authors reported partial ternary phase diagrams, especially isothermal sections at different temperatures.[34,35,37,41,42]

## Experimental Procedure

A Calvet-type microcalorimeter HTMC-1000 (Setaram, Lyon, France), equipped with an automatic drop device for up to 30 drops, was used for the enthalpy of mixing measurements.[38] Control and data evaluation was done with Lab View and HiQ. All measurements were performed under Ar flow (approx. 30 cm^3^/min) in graphite crucibles. The microcalorimeter was calibrated at the end of each measurement series by five additions (approx. 40 mg each) of standard α-Al_2_O_3_ supplied by the National Institute of Standards and Technology (NIST, Gaithersburg, MD, USA). The interval between individual drops was 40 min, the acquisition interval of the heat flow was about 0.5 s. Two thermopiles with more than 200 thermocouples of Pt/Pt-10Rh were used for the determination of the sample temperature (*T*_M_) in the furnace and of the corresponding heat effect for each drop. The measured enthalpy ∆*H*_Signal_ (integrated heat flow at constant pressure) is given by

1 $$\Delta H_{\text{Signal}} = n_{i} (H_{{{\text{m,}}i ,T_{\text{M}} }} - H_{{{\text{m,}}i ,T_{\text{D}} }} ) + \Delta H_{\text{Reaction}} ,$$

where *n*_i_ is the number of moles of the added sample, *H*_m_ denotes molar enthalpies, and *T*_D_ is the drop temperature (room temperature). The molar enthalpy difference $(H_{{{\text{m,}}i ,T_{\text{M}} }} - H_{{{\text{m,}}i ,T_{\text{D}} }} )$ was calculated using the SGTE data for pure elements.[39] Because of the rather small masses added, the partial enthalpies can be given directly as

2 $$\Delta \overline{{H_{i} }} = \frac{{\Delta H_{\text{Reaction}} }}{{n_{i} }},$$

The integral enthalpy of mixing was calculated by summarizing the respective reaction enthalpies and dividing by the total molar amount of substance. The respective binary starting value for each section in the ternary system was calculated from the binary literature data[24,32,40] using the interaction parameters listed in Table 3.

**Fig. 1:**
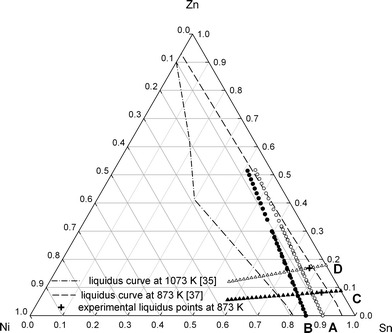
Investigated sections and alloy compositions in the ternary Ni-Sn-Zn system (A and B at 1073 K; C and D at 873 K)

The enthalpy of mixing for ternary liquid Ni-Sn-Zn alloys was determined along two sections at 873 K where pure Ni was dropped into liquid Sn_1−*x*_Zn_*x*_ alloys (*x* = 0.09 and 0.18) as well as along two section at 1073 K where pieces of pure Zn were dropped into liquid Ni_*x*_Sn_1−*x*_ alloys (*x* = 0.10 and 0.15) (Fig. 1). A lower temperature of 873 K was chosen for sections C and D to avoid excessive Zn losses by evaporation. For sections A and B a higher temperature of 1073 K was chosen in order to cover a larger liquid range.

Random errors as well as systematic errors of calorimetry depend on different factors, such as construction of the calorimeter, calibration procedure, signal integration or “chemical errors”, e.g. incomplete reactions or impurities. Considering many calibration measurements done by dropping NIST standard sapphire, the standard deviation can be estimated to be less than ±1%. The systematic errors are mainly caused by parasitic heat flows, base line problems at signal integration and dropping and mixing problems. One can estimate that the random error of the measured enthalpy is about ±150 J.

**Fig. 2:**
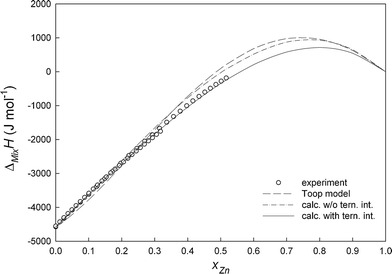
Integral molar enthalpies of mixing of liquid Ni-Sn-Zn alloys along the section *x*_Ni_/*x*_Sn_ ≈ 1:9 at 1073 K; reference states: pure liquid metals

**Fig. 3:**
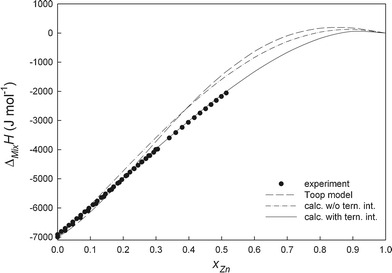
Integral molar enthalpies of mixing of liquid Ni-Sn-Zn alloys along the section *x*_Ni_/*x*_Sn_ ≈ 1:6 at 1073 K; reference states: pure liquid metals

**Fig. 4:**
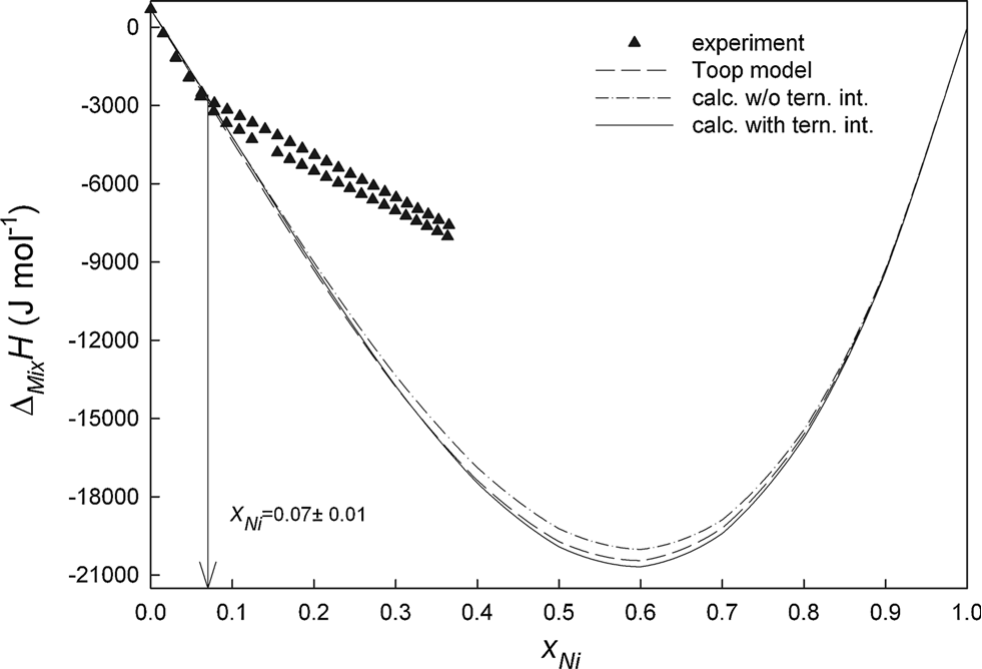
Integral molar enthalpies of mixing of liquid Ni-Sn-Zn alloys along the section *x*_Sn_/*x*_Zn_ ≈ 9:1 at 873 K; reference states: pure liquid metals

All experimental details, i.e. starting amounts, added amounts and resulting heat effects as well as the obtained enthalpy of mixing values are collected in Tables 1 and 2. Figure 2, 3, 4, and 5 show the changes of ∆_Mix_ *H* versus concentration.

**Table 1:** Partial and integral enthalpies of mixing of Ni-Sn-Zn alloys, 1073 K; standard states: pure liquid metals

Dropped mole	Drop enthalpy	Partial enthalpy		Integral enthalpy(a)		
*n*_*i*_, 10^−3^ mol	Δ*H*_Signal_, J mol^-1^	*x*_*i*_(b)	$\Delta \overline{{H_{i} }}$, J mol^-1^	*x*_Zn_	*x*_Sn_	$\Delta_{\text{Mix}} H$, J mol^-1^
*Sect. A: x*_Ni_/*x*_Sn_ ≈ 1:9; *i* = Zn; starting amounts: *n*_Ni_ = 1.8795 × 10^−3^ mol; *n*_Sn_ = 16.9010 × 10^−3^ mol						
0	…	0	…	0	0.8999	−4575
0.4742	35463	0.0123	5404	0.0246	0.8778	−4329
0.9874	35289	0.0373	5230	0.0499	0.8550	−4081
1.5146	34981	0.0623	4922	0.0746	0.8328	−3847
2.0829	34621	0.0872	4562	0.0998	0.8101	−3618
2.6552	34988	0.1119	4929	0.1239	0.7884	−3390
3.2758	35026	0.1362	4967	0.1485	0.7663	−3155
3.9210	34624	0.1606	4564	0.1727	0.7445	−2935
4.6136	35104	0.1850	5044	0.1972	0.7224	−2699
5.3408	34808	0.2093	4749	0.2214	0.7007	−2474
6.1164	34346	0.2335	4287	0.2457	0.6788	−2264
6.9339	34532	0.2577	4472	0.2697	0.6573	−2050
7.7665	34110	0.2811	4050	0.2926	0.6366	−1858
8.6252	34158	0.3036	4099	0.3147	0.6167	−1672
9.5212	34142	0.3256	4083	0.3364	0.5972	−1490
10.4328	33950	0.3468	3891	0.3571	0.5785	−1322
11.3799	33774	0.3672	3715	0.3773	0.5604	−1163
12.3639	33972	0.3871	3913	0.3970	0.5427	−1003
13.3635	33675	0.4064	3616	0.4157	0.5258	−859
14.4186	33334	0.4250	3275	0.4343	0.5091	−728
15.4831	33464	0.4431	3405	0.4519	0.4933	−600
16.5723	33105	0.4603	3046	0.4688	0.4781	−487
17.7193	33126	0.4771	3067	0.4855	0.4630	−376
18.8975	32838	0.4935	2778	0.5016	0.4486	−277
20.0849	32818	0.5092	2758	0.5168	0.4349	−184
*i* = Zn; starting amounts: *n*_Ni_ = 2.8002 × 10^−3^ mol; *n*_Sn_ = 25.3021 × 10^−3^ mol						
0	…	0	…	0	0.9004	−4554
0.3509	35704	0.0062	5594	0.0123	0.8893	−4429
0.7141	35519	0.0186	5409	0.0248	0.8780	−4305
1.0846	35353	0.0310	5243	0.0372	0.8669	−4184
1.4534	34920	0.0432	4810	0.0492	0.8561	−4072
1.8465	35332	0.0554	5222	0.0617	0.8448	−3950
2.2588	34949	0.0680	4839	0.0744	0.8334	−3830
2.6943	35035	0.0809	4925	0.0875	0.8216	−3707
3.1405	35024	0.0940	4914	0.1005	0.8099	−3583
3.6093	34852	0.1072	4742	0.1138	0.7979	−3460
4.0840	34598	0.1204	4488	0.1269	0.7861	−3343
4.5754	34674	0.1335	4564	0.1400	0.7743	−3224
5.0883	34661	0.1467	4551	0.1533	0.7623	−3104
5.6137	34619	0.1599	4509	0.1665	0.7504	−2985
6.1585	34270	0.1731	4160	0.1798	0.7385	−2872
6.7115	33926	0.1863	3816	0.1928	0.7268	−2766
7.2719	34086	0.1992	3975	0.2056	0.7153	−2659
7.8535	34013	0.2120	3902	0.2184	0.7037	−2553
8.4508	34308	0.2248	4198	0.2312	0.6922	−2442
9.0626	33941	0.2375	3830	0.2438	0.6808	−2339
9.6893	33928	0.2501	3818	0.2564	0.6695	−2237
10.3270	33878	0.2626	3768	0.2687	0.6584	−2137
10.9854	33795	0.2749	3685	0.2810	0.6473	−2039
11.6546	33573	0.2871	3463	0.2931	0.6364	−1947
12.3527	33833	0.2992	3722	0.3053	0.6254	−1849
13.0640	33652	0.3113	3541	0.3173	0.6146	−1756
*Sect. B: x*_Ni_/*x*_Sn_ ≈ 1:6; *i* = Zn; starting amounts: *n*_Ni_ = 2.9697 × 10^−3^ mol; *n*_Sn_ = 16.6085 × 10^−3^ mol						
0	…	0	…	0	0.8483	−6998
0.4638	32640	0.0116	2549	0.0231	0.8287	−6777
0.9475	32614	0.0347	2523	0.0462	0.8092	−6558
1.4621	32814	0.0578	2723	0.0695	0.7894	−6331
2.0017	33008	0.0811	2917	0.0928	0.7696	−6100
2.5943	33133	0.1049	3042	0.1170	0.7491	−5855
3.2188	33097	0.1291	3006	0.1412	0.7285	−5613
3.8634	33414	0.1530	3324	0.1648	0.7085	−5367
4.5377	33395	0.1765	3305	0.1882	0.6887	−5124
5.2394	33541	0.1996	3451	0.2111	0.6692	−4882
5.9692	33080	0.2224	2989	0.2337	0.6501	−4657
6.7383	33327	0.2449	3236	0.2560	0.6311	−4427
7.5331	33075	0.2670	2984	0.2779	0.6126	−4209
8.3589	33090	0.2885	2999	0.2992	0.5945	−3996
10.1075	32834	0.3198	2743	0.3405	0.5595	−3599
11.0247	32574	0.3504	2483	0.3602	0.5427	−3417
11.9850	32599	0.3700	2508	0.3797	0.5262	−3237
12.9748	32487	0.3891	2396	0.3986	0.5102	−3065
13.9881	32414	0.4077	2324	0.4167	0.4948	−2903
15.0290	32383	0.4255	2292	0.4343	0.4799	−2746
16.0976	32258	0.4427	2167	0.4512	0.4655	−2599
17.2098	32304	0.4595	2213	0.4678	0.4515	−2454
18.3485	32392	0.4758	2301	0.4838	0.4379	−2311
19.5236	32303	0.4915	2212	0.4993	0.4248	−2175
20.7209	31894	0.5067	1804	0.5142	0.4121	−2057
*i* = Zn; starting amounts: *n*_Co_ = 4.4554 × 10^−3^ mol; *n*_Sn_ = 25.2700 × 10^−3^ mol						
0	…	0	…	0	0.8501	−6915
0.3400	32405	0.0057	2295	0.0113	0.8405	−6811
0.6997	32339	0.0172	2229	0.0230	0.8306	−6704
1.0643	32660	0.0288	2550	0.0346	0.8207	−6594
1.4415	32532	0.0404	2422	0.0463	0.8108	−6485
1.8368	32941	0.0522	2831	0.0582	0.8006	−6369
2.2541	32630	0.0643	2520	0.0705	0.7902	−6253
2.6894	32691	0.0767	2581	0.0830	0.7796	−6134
3.1366	33042	0.0892	2932	0.0954	0.7690	−6011
3.6035	32536	0.1018	2426	0.1081	0.7582	−5892
4.0867	33028	0.1145	2917	0.1209	0.7474	−5766
4.5768	32822	0.1271	2712	0.1334	0.7367	−5645
5.0817	32940	0.1397	2830	0.1460	0.7260	−5522
5.5951	33040	0.1522	2930	0.1584	0.7154	−5400
6.1267	33001	0.1646	2891	0.1709	0.7048	−5277
6.6752	32885	0.1771	2774	0.1834	0.6942	−5155
7.2416	33179	0.1896	3069	0.1959	0.6836	−5029
7.8181	33411	0.2021	3301	0.2082	0.6731	−4901
8.4095	33511	0.2144	3400	0.2205	0.6626	−4773
9.0243	33002	0.2267	2892	0.2329	0.6521	−4651
9.6558	32646	0.2390	2535	0.2452	0.6417	−4536
10.2992	32773	0.2513	2663	0.2573	0.6314	−4420
10.9507	32564	0.2633	2454	0.2692	0.6212	−4310
11.6234	32194	0.2752	2084	0.2811	0.6111	−4206
12.3258	32702	0.2871	2592	0.2931	0.6009	−4092
13.0394	32600.8	0.2990	2491	0.3049	0.5909	−3983

(a) Per mole of mixture

(b) Average value before and after the drop

**Table 2:** Partial and integral enthalpies of mixing of Ni-Sn-Zn alloys, 873 K; standard states: pure liquid metals

Dropped mole	Drop enthalpy	Partial enthalpy		Integral enthalpy(a)		
*n*_i_, 10^−3^ mol	Δ*H*_Signal_, J mol^-1^	*x*_*i*_(b)	$\Delta \overline{{H_{i} }}$, J mol^-1^	*x*_Ni_	*x*_Sn_	$\Delta_{\text{Mix}} H$, J mol^-1^
*Sect. C: x*_Sn_/*x*_Zn_ ≈ 9:1; *i* = Ni; starting amounts: *n*_Sn_ = 25.2346 × 10^−3^ mol; *n*_Zn_ = 2.4245 × 10^−3^ mol						
0	…	0	…	0	0.9123	683
0.4284	−25748	0.0076	−59962	0.0153	0.8984	−242
0.8846	−24490	0.0231	−58704	0.0310	0.8841	−1176
1.3535	−13999	0.0388	−48212	0.0467	0.8698	−1936
1.8391	−3034	0.0545	−37248	0.0623	0.8555	−2518
*2.3383*	*7587*	*0.0701*	*−26627*	*0.0780*	*0.8412*	*−2919*
*2.8598*	*17025*	*0.0858*	*−17189*	*0.0937*	*0.8269*	*−3163*
*3.3915*	*16214*	*0.1015*	*−17999*	*0.1092*	*0.8127*	*−3417*
*3.9359*	*16471*	*0.1169*	*−17743*	*0.1246*	*0.7987*	*−3664*
*4.5032*	*16490*	*0.1323*	*−17724*	*0.1400*	*0.7846*	*−3912*
*5.0829*	*16553*	*0.1476*	*−17661*	*0.1552*	*0.7707*	*−4155*
*5.6891*	*16228*	*0.1629*	*−17986*	*0.1706*	*0.7567*	*−4406*
*6.3094*	*16304*	*0.1782*	*−17910*	*0.1857*	*0.7429*	*−4653*
*6.9459*	*15838*	*0.1932*	*−18376*	*0.2007*	*0.7292*	*−4905*
*7.5905*	*15898*	*0.2080*	*−18316*	*0.2153*	*0.7159*	*−5151*
*8.2575*	*16153*	*0.2226*	*−18061*	*0.2299*	*0.7026*	*−5390*
*8.9571*	*16554*	*0.2373*	*−17660*	*0.2446*	*0.6892*	*−5625*
*9.6625*	*16479*	*0.2518*	*−17735*	*0.2589*	*0.6761*	*−5854*
*10.3824*	*16205*	*0.2659*	*−18009*	*0.2729*	*0.6633*	*−6084*
*11.1205*	*16058*	*0.2798*	*−18155*	*0.2868*	*0.6507*	*−6314*
*11.8841*	*16487*	*0.2936*	*−17727*	*0.3005*	*0.6382*	*−6534*
*12.6560*	*16050*	*0.3072*	*−18164*	*0.3139*	*0.6259*	*−6757*
*13.4374*	*16223*	*0.3204*	*−17991*	*0.3270*	*0.6140*	*−6970*
*14.2431*	*16414*	*0.3334*	*−17800*	*0.3399*	*0.6022*	*−7178*
*15.0654*	*16369*	*0.3463*	*−17844*	*0.3526*	*0.5906*	*−7384*
*15.9136*	*16363*	*0.3589*	*−17851*	*0.3652*	*0.5791*	*−7587*
*i* = Ni; starting amounts: *n*_Sn_ = 25.2818 × 10^−3^ mol; *n*_Zn_ = 2.4438 × 10^−3^ mol						
0	…	0	…	0	0.9119	687
0.4330	−25481	0.0077	−59751	0.0154	0.8978	−242
0.8784	−24916	0.0230	−59186	0.0307	0.8839	−1160
1.8115	−14379	0.0460	−48649	0.0613	0.8559	−2660
2.3156	−2355	0.0692	−36625	0.0771	0.8416	−3230
*2.8352*	*4419*	*0.0849*	*−29851*	*0.0928*	*0.8273*	*−3683*
*3.3701*	*15772*	*0.1006*	*−18497*	*0.1084*	*0.8130*	*−3938*
*3.9239*	*10040*	*0.1162*	*−24229*	*0.1240*	*0.7988*	*−4293*
*5.0895*	*15527*	*0.1395*	*−18742*	*0.1551*	*0.7704*	*−4806*
*5.6884*	*15422*	*0.1627*	*−18848*	*0.1702*	*0.7566*	*−5058*
*6.2987*	*16705*	*0.1777*	*−17564*	*0.1851*	*0.7431*	*−5282*
*6.9339*	*16576*	*0.1926*	*−17694*	*0.2001*	*0.7294*	*−5509*
*7.5933*	*16046*	*0.2075*	*−18223*	*0.2150*	*0.7158*	*−5747*
*8.2679*	*17026*	*0.2223*	*−17244*	*0.2297*	*0.7024*	*−5962*
*8.9444*	*16662*	*0.2368*	*−17607*	*0.2439*	*0.6894*	*−6177*
*9.6405*	*16232*	*0.2510*	*−18037*	*0.2580*	*0.6766*	*−6398*
*10.3527*	*16463*	*0.2649*	*−17806*	*0.2719*	*0.6639*	*−6611*
*11.0981*	*16721*	*0.2789*	*−17548*	*0.2859*	*0.6512*	*−6821*
*11.8519*	*16640*	*0.2927*	*−17630*	*0.2995*	*0.6388*	*−7027*
*12.6078*	*16581*	*0.3060*	*−17689*	*0.3126*	*0.6268*	*−7227*
*13.3807*	*16234*	*0.3191*	*−18036*	*0.3255*	*0.6150*	*−7430*
*14.1806*	*16382*	*0.3320*	*−17887*	*0.3384*	*0.6033*	*−7630*
*15.0078*	*16453*	*0.3448*	*−17817*	*0.3512*	*0.5916*	*−7827*
*15.8386*	*16186*	*0.3574*	*−18083*	*0.3636*	*0.5803*	*−8023*
*Sect. D: x*_Sn_/*x*_Zn_ ≈ 4:1; *i* = Ni; starting amounts: *n*_Sn_ = 25.2954 × 10^−3^ mol; *n*_Zn_ = 5.5993 × 10^−3^ mol						
0	…	0	…	0	0.8188	1409
0.4356	−27712	0.0070	−61936	0.0139	0.8074	528
0.8942	−27644	0.0210	−61867	0.0281	0.7957	−372
2.3401	−1591	0.0493	−35814	0.0704	0.7611	−1914
*2.8627*	*14270*	*0.0776*	*−19953*	*0.0848*	*0.7493*	*−2193*
*3.3958*	*17296*	*0.0919*	*−16928*	*0.0990*	*0.7377*	*−2422*
*3.9385*	*16899*	*0.1060*	*−17325*	*0.1131*	*0.7262*	*−2655*
*4.5007*	*14899*	*0.1201*	*−19325*	*0.1272*	*0.7147*	*−2919*
*5.0752*	*17149*	*0.1341*	*−17074*	*0.1411*	*0.7032*	*−3145*
*5.6794*	*16141*	*0.1482*	*−18083*	*0.1553*	*0.6916*	*−3392*
*6.2922*	*16243*	*0.1622*	*−17980*	*0.1692*	*0.6802*	*−3633*
*6.9180*	*16587*	*0.1761*	*−17636*	*0.1830*	*0.6690*	*−3864*
*7.5767*	*16342*	*0.1899*	*−17881*	*0.1969*	*0.6575*	*−4104*
*8.2482*	*15991*	*0.2038*	*−18232*	*0.2107*	*0.6462*	*−4347*
*8.9409*	*16355*	*0.2176*	*−17869*	*0.2244*	*0.6350*	*−4582*
*9.6419*	*16651*	*0.2312*	*−17572*	*0.2379*	*0.6240*	*−4806*
*10.3848*	*16676*	*0.2447*	*−17547*	*0.2516*	*0.6128*	*−5036*
*11.1370*	*16680*	*0.2583*	*−17543*	*0.2650*	*0.6018*	*−5260*
*11.9000*	*16546*	*0.2715*	*−17678*	*0.2781*	*0.5911*	*−5481*
*12.6854*	*16366*	*0.2846*	*−17857*	*0.2911*	*0.5804*	*−5704*
*13.4884*	*16751*	*0.2975*	*−17472*	*0.3039*	*0.5699*	*−5917*
*14.3170*	*16507*	*0.3103*	*−17716*	*0.3167*	*0.5595*	*−6133*
*15.1782*	*16826*	*0.3231*	*−17397*	*0.3294*	*0.5490*	*−6344*
*i* = Ni; starting amounts: *n*_Sn_ = 25.2995 × 10^−3^ mol; *n*_Zn_ = 5.5168 × 10^−3^ mol						
0	…	0	…	0	0.8210	1392
0.4360	−29183	0.0070	−63435	0.0140	0.8095	488
0.8924	−28183	0.0210	−62434	0.0281	0.7979	−418
1.3610	−27581	0.0352	−61833	0.0423	0.7863	−1312
1.8353	−15096	0.0493	−49347	0.0562	0.7748	−2010
*2.3273*	*−10944*	*0.0632*	*−45196*	*0.0702*	*0.7633*	*−2651*
*2.8471*	*14858*	*0.0774*	*−19394*	*0.0846*	*0.7515*	*−2910*
*3.3788*	*16836*	*0.0917*	*−17416*	*0.0988*	*0.7399*	*−3135*
*3.9352*	*16461*	*0.1060*	*−17790*	*0.1132*	*0.7280*	*−3370*
*4.5002*	*16638*	*0.1203*	*−17614*	*0.1274*	*0.7164*	*−3598*
*5.0793*	*16517*	*0.1345*	*−17734*	*0.1415*	*0.7048*	*−3826*
*5.6901*	*15904*	*0.1487*	*−18347*	*0.1559*	*0.6930*	*−4069*
*6.3075*	*16388*	*0.1629*	*−17864*	*0.1699*	*0.6815*	*−4298*
*6.9417*	*16140*	*0.1769*	*−18112*	*0.1838*	*0.6700*	*−4530*
*7.5937*	*16510*	*0.1908*	*−17741*	*0.1977*	*0.6587*	*−4755*
*8.2566*	*16364*	*0.2045*	*−17888*	*0.2113*	*0.6475*	*−4977*
*8.9334*	*15615*	*0.2180*	*−18637*	*0.2247*	*0.6365*	*−5210*
*9.6490*	*16360*	*0.2316*	*−17892*	*0.2385*	*0.6252*	*−5434*
*10.3765*	*16451*	*0.2452*	*−17801*	*0.2519*	*0.6142*	*−5653*
*11.1069*	*17586*	*0.2584*	*−16666*	*0.2649*	*0.6035*	*−5845*
*11.8574*	*16722*	*0.2714*	*−17530*	*0.2779*	*0.5929*	*−6050*
*12.6251*	*17009*	*0.2842*	*−17242*	*0.2906*	*0.5824*	*−6248*
*13.4052*	*16959*	*0.2969*	*−17293*	*0.3031*	*0.5721*	*−6443*
*14.1947*	*16430*	*0.3092*	*−17822*	*0.3154*	*0.5621*	*−6642*
*15.0158*	*16178*	*0.3215*	*−18073*	*0.3276*	*0.5520*	*−6847*
*15.8574*	*16070*	*0.3337*	*−18182*	*0.3398*	*0.5421*	*−7051*

(a) Per mole of mixture

(b) Average value before and after the drop

**Fig. 5:**
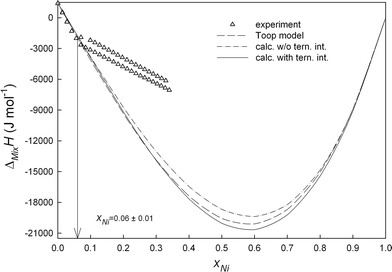
Integral molar enthalpies of mixing of liquid Ni-Sn-Zn alloys along the section *x*_Sn_/*x*_Zn_ ≈ 4:1 at 873 K; reference states: pure liquid metals

## Results and Discussion

### Experimental Results

According to the phase equilibria at 1073 K,[35] the experimental temperature for (Ni-Sn) + Zn alloys along the sections A (*x*_Ni_/*x*_Sn_ ≈ 1:9) and B (*x*_Ni_/*x*_Sn_ ≈ 1:6) was high enough to obtain completely liquid alloys over the entire investigated concentration range; see dashed-points line in Fig. 1. In contrary, the clear kinks in the enthalpy curves for (Sn-Zn) + Ni alloys along the cross sections C (*x*_Sn_/*x*_Zn_ = 9:1) and D (*x*_Sn_/*x*_Zn_ ≈ 4:1) shown in Fig. 4 and 5 indicate formation of a solid phase and denote the liquidus limit at 873 K. The corresponding points are in reasonable agreement with the estimated liquidus line at 873 K given by Yuan et al.[37] which is shown as a dashed line in Fig. 1. Accordingly, the values within the italicized values in Table 2 are for alloys beyond the liquidus limit.

The obtained enthalpies of mixing are exothermic along all sections, indicating the preferred interactions between unlike kinds of atoms. It should be noted that the enthalpy of mixing data for both (Sn-Zn) + Ni alloys are practically identical in the concentration range after formation of a solid phase. This may be explained by formation of the same phase in both cases.

### Ternary Modeling

The interaction parameters of the binary systems were taken directly from the literature[24,32,40] and are listed in Table 3. The enthalpy of mixing for the ternary system was treated by a least-squares fit using the following Redlich-Kister-Muggianu polynomial,[9] which takes into account additional ternary interactions:

3 $$\Delta_{\text{Mix}} H = \sum\limits_{i} {\sum\limits_{j > i} {\left[ {x_{i} x_{j} \sum\limits_{\upnu } {{}^{\upnu }L_{i:j} \left( {x_{i} - x_{j} } \right)}^{\upnu } } \right]} } + x_{i} x_{j} x_{k} \left( {{}^{0}M_{i:j:k} x_{i} + {}^{1}M_{i:j:k} x_{j} + {}^{2}M_{i:j:k} x_{k} } \right),$$

**Table 3:** Binary and ternary interaction parameters for Ni-Sn-Zn

System	Interaction parameters, J mol^-1^	Reference
Ni-Sn	^0^L = −80659 + 183*T ^1^L = −24617 − 953*T	[24]
Ni-Zn	^0^L = −50722 ^1^L = 8436 ^2^L = −25136	[32]
Sn-Zn	^0^L = 12728 ^1^L = −5074	[40]
Ni-Sn-Zn	^0^M = −156468 ^1^M = 26414 ^2^M = −64909	Present work

Temperatures (*T*) in kelvin

where *i*, *j*, *k* are equal to 1, 2, 3 for the elements Ni, Sn and Zn respectively; ^*ν*^ *L*_*i:j*_ (*ν* = 0, 1, 2,…) are the interaction parameters of the three binary systems; ^*ν*^ *M*_*i:j:k*_ (*ν* = 0, 1, 2) are three ternary interaction parameters; *x*_*i*_, *x*_*j*_, *x*_*k*_ are the corresponding mole fractions. The enthalpy of mixing is temperature independent for the two binary systems Ni-Zn and Sn-Zn, and it shows small temperature dependence for the Ni-Sn system. Therefore, any possible temperature dependence of Δ_Mix_ *H* in the ternary Ni-Sn-Zn system was neglected in the present evaluation. The parameters ^*ν*^ *M*_*i:j:k*_, obtained from the experimental enthalpy of mixing data, represent the additional contribution due to ternary interactions (Table 3). The difference between experimental and calculated enthalpy of mixing data is not more than ±250 J mol^-1^ which is within the limits of the experimental errors. This can be seen from Fig. 2, 3, 4, and 5 where full lines refer to calculated values with ternary interaction, dashed lines to those without.

As an alternative, the so-called Toop model[10] was used to calculate the ternary enthalpy values. This model uses an asymmetric extrapolation to predict ternary thermodynamic quantities based on binary data. The corresponding equation is:

4 $$\begin{aligned} \Delta_{\text{Mix}} H = & \frac{{x_{j} }}{1 - xi}\Delta_{\text{Mix}} H_{i,j} \left( {x_{i} ,1 - x_{i} } \right) + \frac{{x_{k} }}{{1 - x_{i} }}\Delta_{\text{Mix}} H_{i,k} \left( {x_{i} ,1 - x_{i} } \right) \\ & + \left( {x_{j} + x_{k} } \right)^{2} \Delta_{\text{Mix}} H_{j,k} \left( {\frac{{x_{j} }}{{x_{j} + x_{k} }},\frac{{x_{k} }}{{x_{j} + x_{k} }}} \right), \\ \end{aligned}$$

where ∆_Mix_ *H*_*i,j*_, ∆_Mix_ *H*_*i,k*_, and ∆_Mix_ *H*_*j,k*_, are the enthalpies of mixing for liquid Ni-Sn, Ni-Zn and Sn-Zn alloys, respectively. The enthalpies of mixing values of binary sub-systems were calculated by a Redlich-Kister polynomial based on interaction parameters from the literature given in Table 3.

A comparison of the experimental enthalpy of mixing with the calculated data along all investigated cross sections is shown in Fig. 2, 3, 4, and 5. It can be seen that the calculated curves based on the Toop model are in good agreement with our fitting without ternary interaction terms and differ from the experimental data by less than 400 J mol^-1^ except for the section *x*_Ni_/*x*_Sn_ ≈ 1:9 where the deviation is higher. This comparatively small improvement of the fits adding ternary interaction terms, however, is not a proof for the existence of real ternary interaction in the liquid phase. Both, the Muggianu- and the Toop-model for the extrapolation of binary enthalpy data into the ternary are of limiting significance. Thus the ternary terms could also compensate shortcomings of the binary extrapolations models

**Fig. 6:**
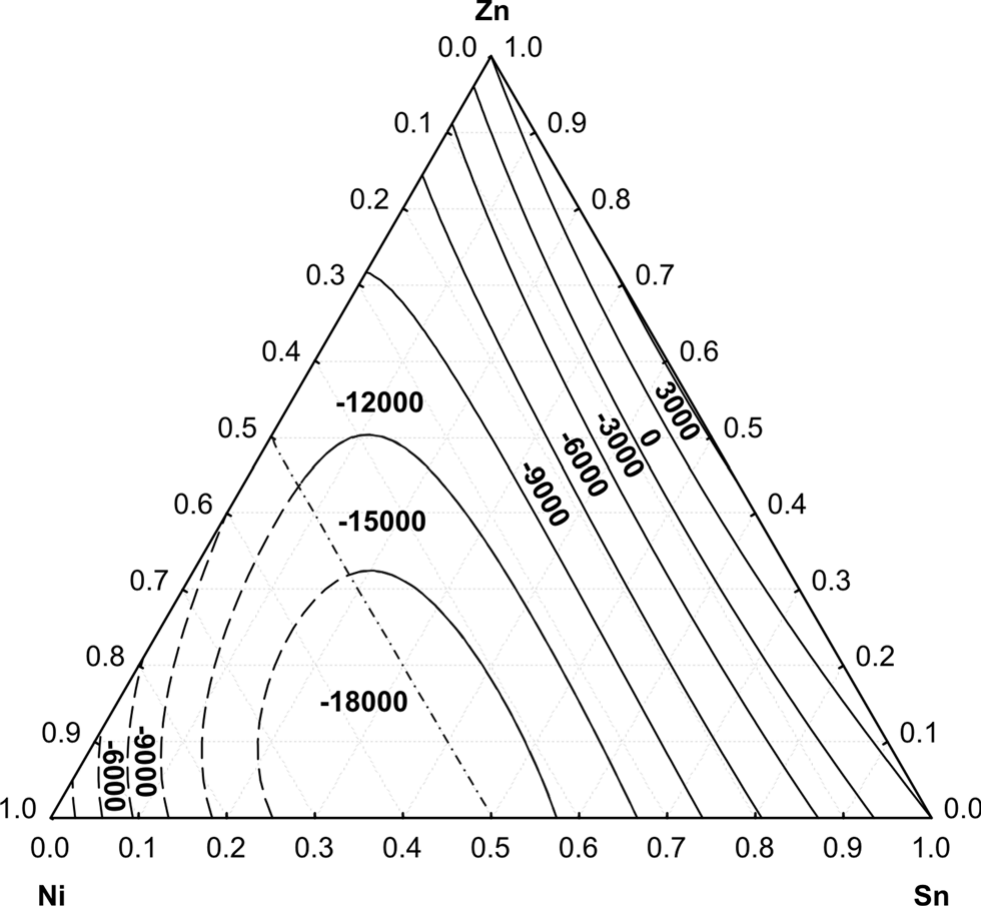
Isoenthalpy curves of liquid Ni-Sn-Zn alloys valid for the temperature range 873-1073 K; reference states: pure liquid metals, numbers given in J mol^-1^

Finally, an iso-enthalpy plot is presented in Fig. 6. The values are exothermic in most of the ternary composition range, except close to the binary Sn-Zn system. The minimum values are actually in the binary Ni-Sn system. All data beyond the liquidus limit are considered as enthalpy of mixing of the metastable liquid.

## Conclusions

Enthalpies of mixing in the liquid Ni-Sn-Zn system were measured along four sections using a high temperature Calvet microcalorimeter. For two sections *x*_Ni_/*x*_Sn_ ≈ 1:9, *x*_Ni_/*x*_Sn_ ≈ 1:6 were measured at 1073 K, for the other two sections, i.e. *x*_Sn_/*x*_Zn_ ≈ 9:1, *x*_Sn_/*x*_Zn_ ≈ 4:1, experiments were performed at 873 K. A comparison of experimental and calculated enthalpy of mixing values based on Redlich-Kister-Muggianu data fits and on the Toop extrapolation model shows good agreement.

Based on the experimental data three ternary interaction parameters ^*ν*^ *M*_*i:j:k*_ were obtained according to the Redlich-Kister-Muggianu polynomial. These data could be used in a standard CALPHAD procedure for the assessment of the equilibrium phase diagram.
